# Supplementary material for: Hydrogen-bearing vesicles in space weathered lunar calcium-phosphates
Source: Commun Earth Environ. 2023 Nov 15;4(1):414. doi: 10.1038/s43247-023-01060-5 (PMC11041702; doi:10.1038/s43247-023-01060-5)
Supplement: Supplementary file 1 — Supplementary Information [file 43247_2023_1060_MOESM1_ESM.pdf]

Supplementary Material

Hydrogen-bearing vesicles in space weathered lunar calcium-phosphates

Katherine D. Burgess[1*], Brittany A. Cymes[1,2], and Rhonda M. Stroud[1,3]

[1]Materials Science and Technology Division, U.S. Naval Research Laboratory, Washington, DC, USA;
[2]Jacobs, NASA Johnson Space Center, Houston, TX, USA;
[3]School of Earth and Space Exploration, Arizona State University, Tempe, AZ 85287, USA

* Corresponding author: kate.burgess@nrl.navy.mil

Table S1. Average composition of Ca-phosphates (at%) as determined by STEM-EDS.
Figure S1. HAADF image and EDS maps of apatite at large field of view.
Figure S2. Representative EDS spectra from apatite and merrillite grains.
Figure S3. Space weathering context of merrillite adjacent to agglutinitic glass.
Figure S4. Vesicular, $npFe^0$-rich splash melt on apatite with EDS and EELS.
Figure S5. Alignment of vesicles in apatite.
Figure S6. Effect of dwell time and repeated measurements on ~13 eV peak in apatite.

Table S1. Average composition of Ca-phosphates (at%) as determined by STEM-EDS. OH is calculated on a per formula basis (p.f.u.) based on measured concentration of F, Cl, and Ca.

	**average apatite**	**1σ (%)**	**average merrillite**	**1σ (%)**
**C**	0.99	8.9	0.00	-
**O**	58.60	3.1	65.61	3.1
**F**	2.90	4.0	0.15	52.1
**Na**	0.01	25.1	0.15	23.9
**Mg**	0.12	39.3	1.80	5.5
**Al**	0.26	28.9	0.18	15.1
**Si**	1.02	2.1	0.25	19.7
**P**	12.38	3.2	12.31	3.3
**S**	0.05	5.3	0.19	20.2
**Cl**	0.12	15.1	0.21	19.5
**Ca**	22.88	3.1	17.82	3.2
**Fe**	0.22	7.6	0.26	12.4
**Y**	0.29	4.5	0.45	9.6
**La**	0.02	20.8	0.07	19.2
**Ce**	0.08	12.6	0.36	12.8
**Nd**	0.09	12.5	0.22	17.2
**p.f.u**				
**F**	0.63			
**Cl**	0.03			
**OH**	0.34			

Figure S1.

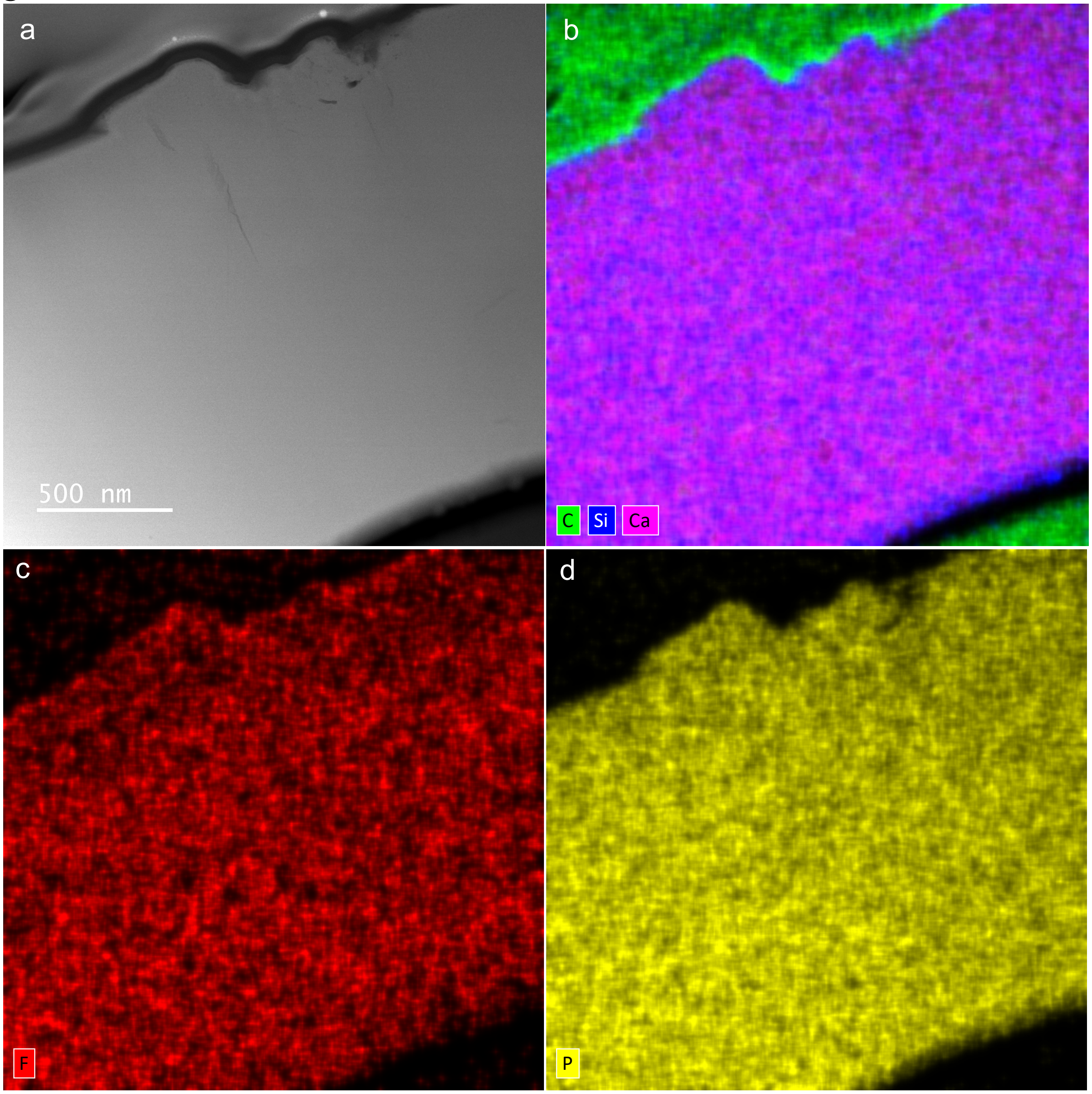

Figure S1. HAADF image and EDS maps showing larger field of view of apatite used in composition calculation. The apatite composition is uniform across the sample except for a thin 10-20 nm rim and very thin Si-rich vapor deposited coating. The apatite is F-rich.

Figure S2.

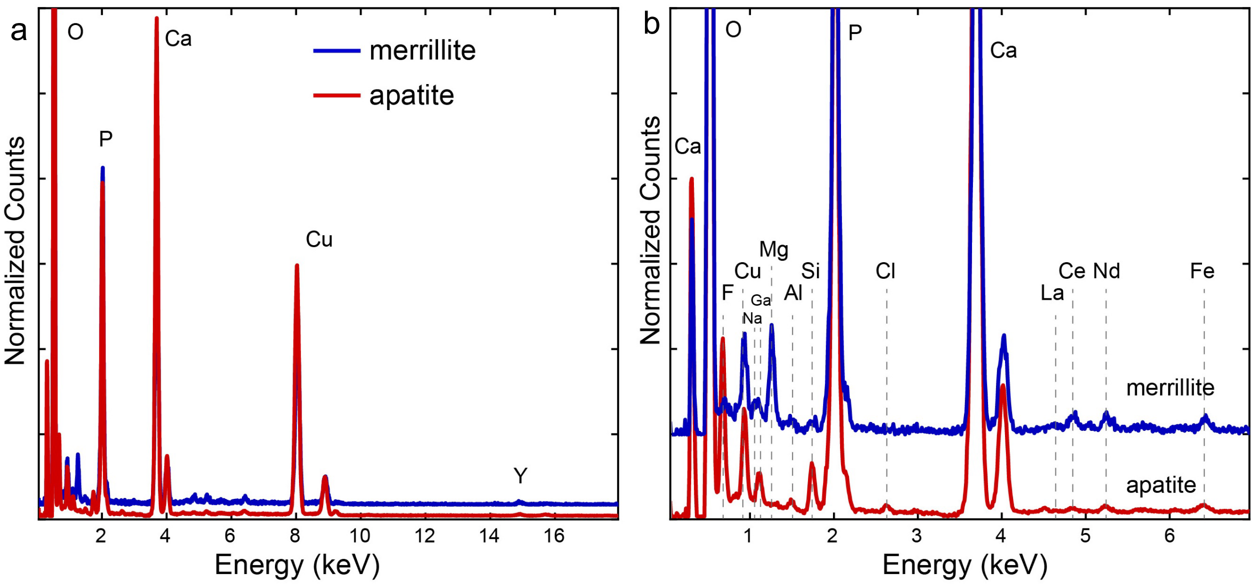

Figure S2. Representative EDS spectra from apatite and merrillite grains. (a) Spectra showing a small Y peak in merrillite and relative peak heights for major and minor elements. Spectra were normalized to O then offset vertically for clarity. A small portion of the O peak is cut off at the top. Cu is present in the sample grid and microscope pole piece. (b) Zoomed in view in energy and counts to highlight peaks for REE and other minor elements. Minor Ga is from FIB preparation.

Figure S3.

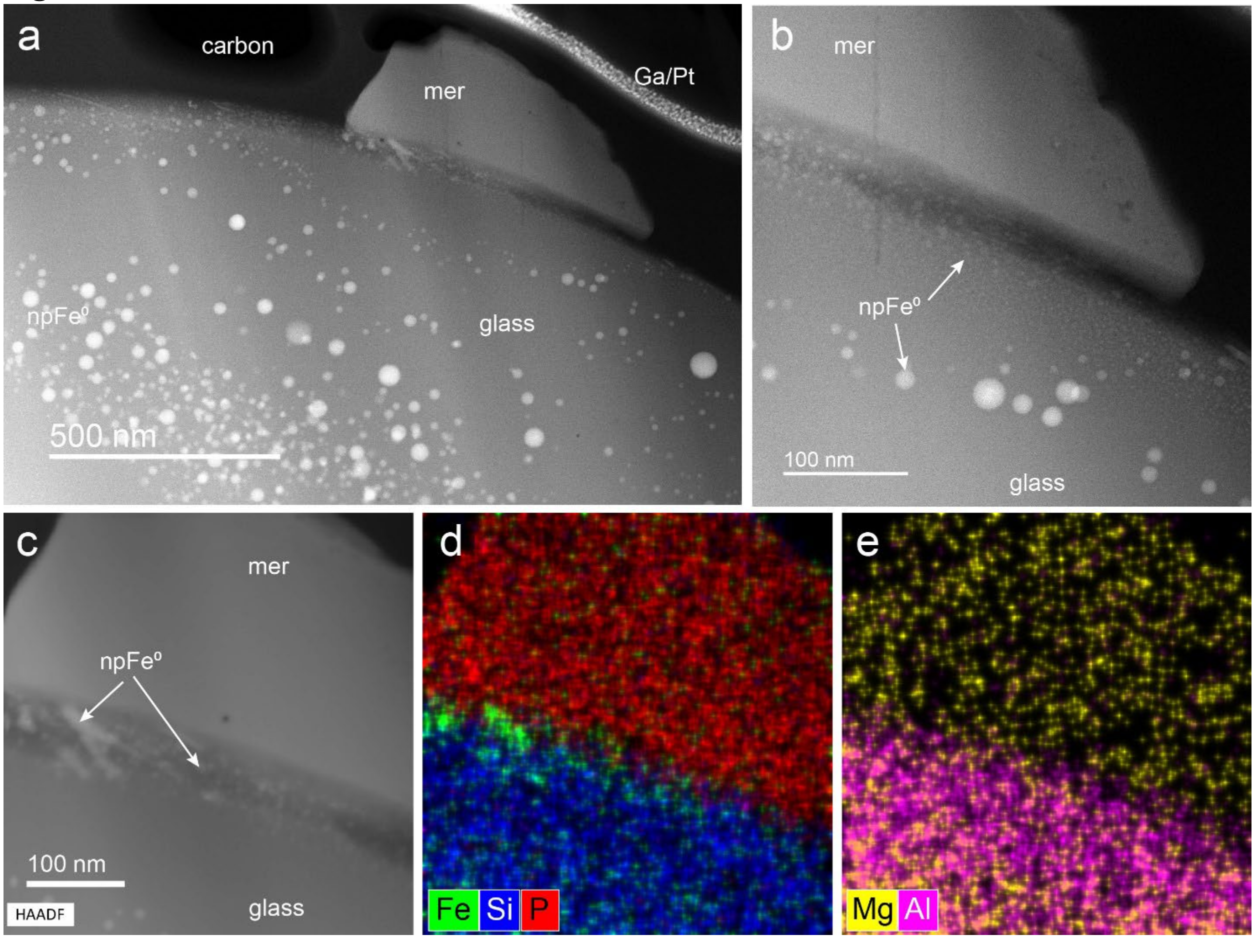

Figure S3. Space weathering context of merrillite adjacent to agglutinitic glass. (a-c) Intermediate field-of-view HAADF images showing concentration of $npFe^0$ in rim of silicate glass beneath and extending beyond the merrillite grain. Mer = merrillite. (d,e) EDS maps of merrillite-glass interface in (c) showing $npFe^0$ and variability in glass composition.

Figure S4.

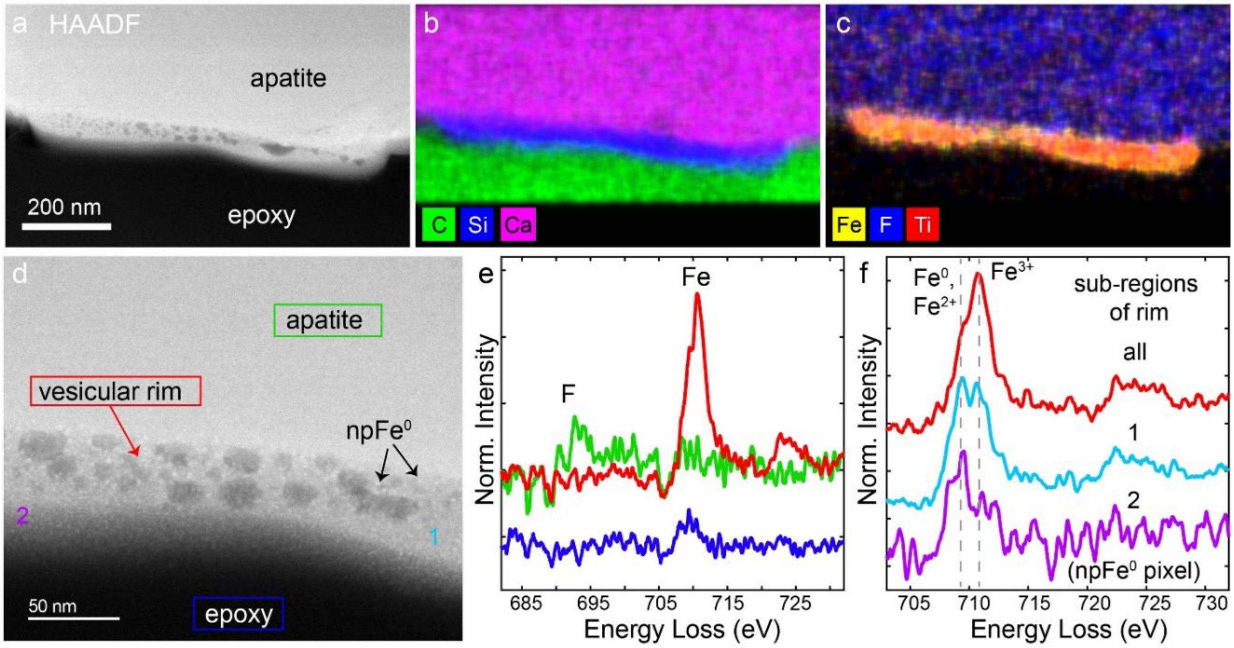

Figure S4. Vesicular, $npFe^0$-rich splash melt on apatite. (a) HAADF image showing a vesicular, $npFe^0$-rich, silicate rim along the bottom of apatite grain. The silicate melt splash is amorphous and contains the major elements expected in lunar samples (i.e., Si, Al, Ca, Mg, Fe, Ti, O) although the composition is not uniform across the ~800 nm coating. The coating contains vesicles with a range of sizes, ~5 nm to greater than 60 nm, with larger vesicles present in the right portion. There are very small vesicles within the apatite where it is in contact with the right side of the silicate rim, although unfortunately beam damage precluded clear measurements of this material. (b-c) Semi-quantitative EDS element maps showing uniform apatite composition toward rim and Fe and Ti-rich silicate rim. (d) HAADF image showing detail of rim and regions summed for spectral acquisitions. (e) EEL spectra from apatite (green), silicate rim (red), and carbon epoxy (blue). (f) EEL spectra from all of rim compared to small ROIs (1, 2) within the silicate rim showing Fe oxidation is variable, with metallic nanoparticles in an oxidized matrix. The right-most portion of the silicate coating is overall less oxidized than the left portion, although individual pixels throughout the rim display signatures of metallic Fe.

Figure S5.

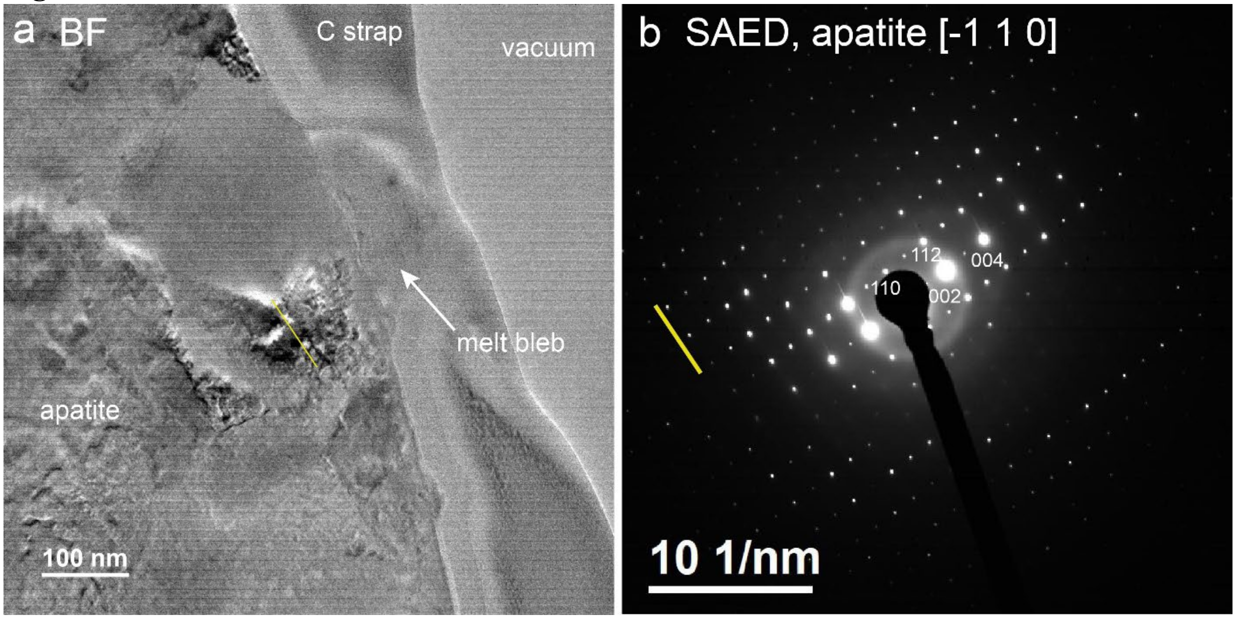

Figure S5. Alignment of vesicles in apatite. (a) Bright field image from JEOL2200FS showing apatite and melt bleb. Image is oriented ~90° relative to Fig. 3 and 4. (b) Selected area diffraction pattern of the apatite along the [1 -1 2] zone axis. Yellow line drawn parallel to vesicles in the apatite shows the vesicles lie along the (001) plane.

Figure S6.

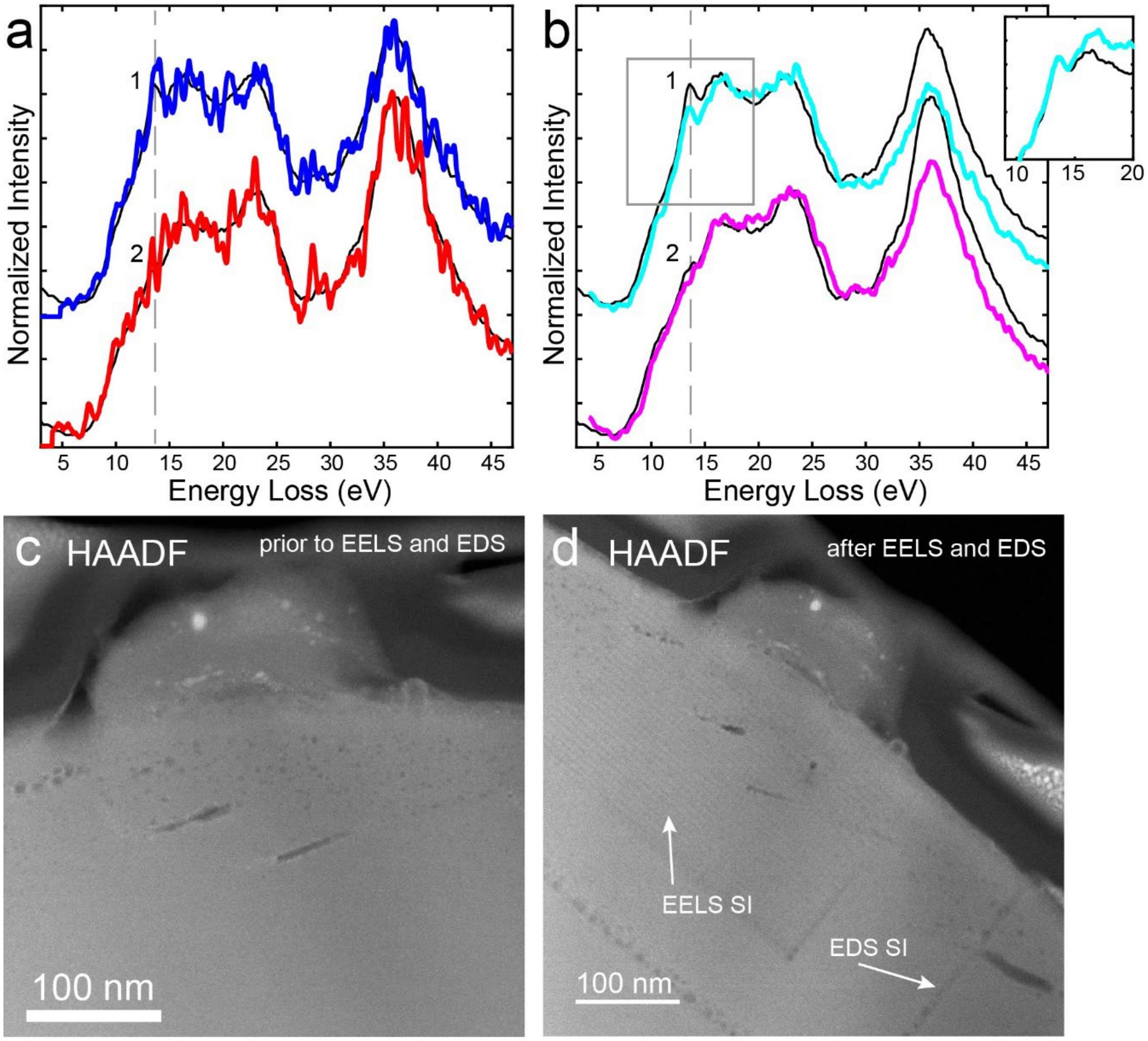

Figure S6. Effect of dwell time and repeated measurements on 13 eV peak in apatite. (a) Spectra 1 & 2 from vesicle in apatite collected using two different dwell times. Thick blue and red lines used dwell time 0.1 ms/pixel and thinner black lines used 5 ms/pixel. Thin lines are shown in Fig. 5 in manuscript. Spectra with short dwell times are much noisier but clearly show presence and similar intensity of 13 eV peak indicating presence of H within the vesicle (spectrum 1) but not below the vesicle (spectrum 2). (b) Spectra 1 & 2 (black lines) compared to spectra collected from within and next to the same vesicle in the same instrument six months later, after several longer dwell-time maps were collected (i.e., O data in Fig. 5 at 50 ms/pixel). The shape of the spectra at energy loss greater than ~30 eV is altered, possibly due to Ca loss, but the 13 eV peak is present. The inset shows that the intensity of the peak is similar or slightly decreased depending on assumptions about how the background should be normalized. (c) HAADF image of the region prior to EELS and EDS measurements, same as in Fig. 5a. (d) HAADF image after EDS and EELS from (b). Edges of the scan boxes are apparent due to beam damage, and individual dwell spots are seen from EELS with 50 ms/pixel. Vesicles do not alter shape or size due to scans.
